# Acupotomy by ultrasound-guided versus anatomical guidance in knee osteoarthritis: A protocol for systematic review and meta-analysis

**DOI:** 10.1097/MD.0000000000031693

**Published:** 2022-11-25

**Authors:** Li Wang, Jiantong Wei, Zhi Qian, Jun Qian

**Affiliations:** a Department of Ultrasound, Zhangye People’s Hospital Affiliated to Hexi University, Gansu, China; b Department of Orthopedics, Zhangye People’s Hospital Affiliated to Hexi University, Gansu, China.

**Keywords:** knee osteoarthritis, pain, range of motion, ultrasonic-guided acupotomy

## Abstract

At present, there is no systematic evaluation on whether ultrasonic-guided acupotomy is more effective compared with anatomical guidance in knee osteoarthritis. We conducted a protocol for systematic review and meta-analysis to provide a method for evaluating the effectiveness and safety of acupotomy by ultrasound-guided technique.

**Methods:**

An all-round retrieval will be performed in the following electronic journal databases from their inception to October 2022, which comprise PubMed, MEDLINE, EMBASE, Cochrane Library, China National Knowledge Infrastructure, Wanfang data, Chinese Scientific Journals Database, and China biomedical literature database. The following key words were used on combination with Boolean operators AND or OR: “acupotomy,” “ultrasound,” “knee osteoarthritis.” Two authors completed the quality assessment using the Cochrane Collaborations risk of bias tool. The meta-analysis was conducted using Review Manager 5.3 software from the Cochrane Collaboration (London, UK).

**Results:**

The findings of this study will be submitted to peer-reviewed journals for publication.

**Conclusion:**

This systematic review will provide evidence to judge whether acupotomy by ultrasound-guided technique is effective and safe for knee osteoarthritis.

## 1. Introduction

Knee osteoarthritis (OA) is the most prevalent chronic joint disease.^[1–3]^ Cartilage is the central tissue affected by OA and causes subsequent symptoms, including joint pain, stiffness and joint swelling, which diminishes the range of motion.^[4,5]^ It is one of the major causes of deformity, resulting in huge medical expense and poor quality of life. It affects nearly 34% of those aged 65 and older.^[6]^ The number of patients with knee OA has increased in tandem with population aging and it remains a huge healthcare challenge. Currently, no reliable treatment has been confirmed to prevent progression of knee OA. The aim of treatment was to relieve pain and increase functional outcomes.^[7,8]^ Numerous conservative methods for pain management, including modification of daily activities and peri-articular infiltration analgesia have been tested, and the optimal method is currently still under debate.

Acupotomy therapy is widely used in Chinese clinical practice and recommended in the Chinese medicine expert consensus for knee OA.^[9]^ Acupotomy is a type of acupuncture used in traditional Chinese medicine (TCM), and it has both the characteristics of a “needle” in traditional Chinese medicine and a “knife” in Western medicine.^[10]^ The mechanism of acupotomy remains unclear and remains to be explored, but acupotomy can be used to release ligaments, joint sacs, and synovium.^[11]^ Some studies have shown that acupotomy therapy can release adhesions, alter the mechanical balance of the knee joint, improve lymphatic circulation, and reduce abnormal tissue pressures.^[12]^ Acupotomy has been widely used clinically with a satisfactory efficacy. With the development of ultrasound technology, ultrasound-guided acupotomy has shown great value in clinical practice.^[13,14]^ But it is not yet clear that ultrasound-guided acupotomy is effective and safe in knee OA. Therefore, it is important to reevaluate the available evidence to reach a relatively convincing conclusion that acupotomy by ultrasound-guided technique is a better choice than anatomical guidance.

## 2. Methods

### 2.1. Protocol and registration

This protocol was drafted and reported in accordance with the Preferred Reporting Items for Systematic Reviews and Meta-Analyses Protocols (PRISMA-P) guidelines.^[15]^ If there are any adjustments throughout the study, we will fix and update the details in the final report. The review protocol was registered with the International Prospective Register of Systematic Reviews (PROSPERO),^[16]^ registration number (CRD42019145167).

### 2.2. Ethics

We will not need individual data of each patient in the research as this is a systematic review. Therefore institutional review board approval and ethics committee is not needed.

### 2.3. Inclusion criteria

#### 2.3.1. Types of studies.

All randomized controlled trials (RCTs) comparing acupotomy by ultrasound-guided and anatomical guidance will be included without any restriction to publication status or language. Non-randomized controlled trial and uncontrolled clinical trials will be excluded. Any study with a sample size of <10 people will also be excluded from this review.

#### 2.3.2. Types of patients.

All patients undergoing acupotomy by the ultrasound-guided technique and anatomical guidance will be included in the trial. All eligible patients will not be restricted by disease, age, sex, race, education, or economic status. Patients who will be decided unsuitable for acupotomy by ultrasound-guided technique, such as patients with fracture and dislocation, space-occupying lesions, cardiovascular and cerebrovascular diseases and other serious diseases will be excluded.

#### 2.3.3. Types of interventions.

The treatment group will be treated with acupotomy by ultrasound-guided technique (there is no limit on the needle materials, ultrasonic equipment, and course of treatment). The control group will adapt to routine acupotomy. Studies comparing different acupotomy insertion sites or different forms of acupotomy will be excluded.

#### 2.3.4. Outcome measures.

The primary outcomes were visual analogue score,^[17]^ Western Ontario and McMaster Universities (WOMAC) index^[18]^ and range of motion. Secondary outcomes included quality of life and adverse effects.

### 2.4. Search strategy

Following databases will be searched: PubMed, MEDLINE, EMBASE, Cochrane Library, China National Knowledge Infrastructure, Wanfang data, Chinese Scientific Journals Database, and China biomedical literature database. We will select the eligible studies published up to October, 2022. We adopt the combination of heading terms and free words as a search strategy which is decided by all the reviewers. Search terms: acupotomy, ultrasound, knee osteoarthritis. Taking PubMed as an example, the initial search strategy is shown in Table 1, which will be adjusted according to the specific database. The reference lists of the included studies were also checked for additional studies that were not identified with the database search. The flowchart of this systematic review is shown in Figure 1.

**Table 1:** Search strategy of PubMed.

#1 “random*”[Text Word] OR allocation[Text Word] OR “random allocation”[Text Word] OR placebo[Text Word] OR single blind[Text Word] OR double blind[Text Word] OR “randomized controlled trial*”[Text Word] OR RCT[Text Word]
#2 randomized controlled trial[Publication Type]
#3 #1 OR #2
#4 animals NOT humans
#5 #3 NOT #4
#6 “acupotomy” [Text Word] OR “ needle scalpel “[Text Word] OR needle knife [Text Word]
#7 Osteoarthritis, Knee [MeSH] OR “Knee Osteoarthritides”[Title/Abstract] OR “Knee Osteoarthritis” [Title/Abstract] OR “Osteoarthritides, Knee” [Title/Abstract]
#8 “ultrasonic guidance” [Text Word] OR “ultrasonic-guide “[Text Word]
#9 “anatomical guidance” [Text Word] OR “landmark “[Text Word] OR “non-ultrasound guidance”
#10 #5 AND #6 AND #7 AND #8 AND #9

**Figure 1.**
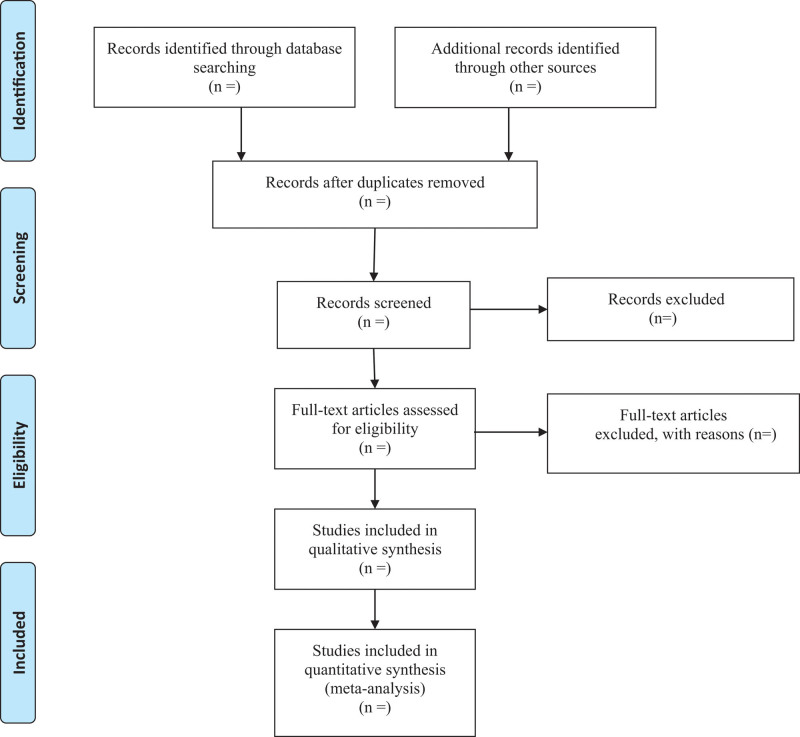
Flow diagram of study selection.

### 2.5. Data extraction

Two independent authors will extract the below descriptive information from the included articles: demographic information of patients, such as average age, number of patients, sex ratio and body mass index; study characteristics, such as authors, year of publication, study language, study design, and the average follow-up period; details of interventions and outcome measures. If the data cannot be directly extracted or is missing, we will contact the relevant author to ensure that the information is complete.

### 2.6. Risk of bias

The risk of bias assessment of the included studies was performed by two authors independently using the Cochrane Collaborations risk of bias tool.^[19]^ This tool included seven aspects which were sequence generation (selection bias), allocation sequence concealment (selection bias), blinding of participants and personnel (performance bias), blinding of outcome assessment (detection bias), incomplete outcome data (attrition bias), selective outcome reporting (reporting bias) and other bias (baseline balance and fund). Additionally, each of the aspects was ranked low risk of bias, high risk of bias, and unclear risk of bias.

### 2.7. Statistical analysis

We performed the meta-analysis by Review Manager software version 5.3 from the Cochrane Collaboration (London, UK). Continuous variables were expressed as the weighted mean difference or standardized mean difference and 95% confidence interval. Weighted mean difference was used when data were measured in the same scale and standardized mean difference were used if data were measured using different scales. Heterogeneity among the studies was quantified with the *I*^2^ statistic. If *I*^2^ > 50% or *P* < .1, a random-effect model was used to decrease heterogeneity, and the subgroup and sensitivity analysis were performed to explore the sources of heterogeneity; otherwise, heterogeneity was negligible and a fixed-effect model was used. To evaluate publication bias, we perform a funnel plot if the number of included studies is sufficient (>10 articles). A symmetrical funnel plot indicates no possibility of publication bias, while an asymmetrical funnel plot indicates a high possibility of publication bias.^[20]^

## 3. Discussion

The purpose of this study is to evaluate whether ultrasonic-guided acupotomy is more effective compared with anatomical guidance. The conclusions drawn from this review may benefit patients who are ready for acupotomy, as well as clinicians. This review has some potential limitations:

1. different types of needle knives, ultrasound equipment, and diseases may lead to heterogeneities;
2. the quality of the included studies may be poor, which will decrease the evidence level;
3. most of the studies are reported in Chinese, which may produce language bias.

At present, there is no systematic evaluation or research undergoing on this issue. Therefore, it is important to perform a systematic review to reach a relatively convincing conclusion that ultrasound-guided technique is a better choice for acupotomy.

## Author contributions

**Data analysis**: Jiantong Wei.

**Data collection**: Zhi Qian.

**Data curation**: Jiantong Wei.

**Investigation methodology**: Zhi Qian.

**Study design**: Jun Qian.

**Writing – original draft**: Li Wang.

**Writing – review & editing**: Jun Qian.
